# GP perspectives of irritable bowel syndrome – an accepted illness, but management deviates from guidelines: a qualitative study

**DOI:** 10.1186/1471-2296-14-92

**Published:** 2013-06-27

**Authors:** Elaine F Harkness, Val Harrington, Sue Hinder, Sarah J O’Brien, David G Thompson, Paula Beech, Carolyn A Chew-Graham

**Affiliations:** 1 Institute of Inflammation and Repair, Stopford Building, University of Manchester, Oxford Road, Manchester M13 9PL, England; 2 Centre for the History of Science Technology & Medicine, Simon Building, University of Manchester, Oxford Road, Manchester M13 9PL, England; 3 RaFT Research and Consulting-Asking the Right Questions, Lower Hall, Downham, Clitheroe, Lancashire BB7 4BN, England; 4 Institute of Infection and Global Health, Leahurst Campus, University of Liverpool, Chester High Road, Neston, South Wirral CH64 7TE, England; 5 Gastrointestinal Centre, Institute of Inflammation & Repair, University of Manchester, Clinical Sciences Building, Salford Royal NHS Foundation Trust, Stott Lane, Salford M6 8HD, England; 6 Research Institute, Primary Care and Health Sciences, Keele University, Keele, Staffordshire ST5 5BG, England

**Keywords:** Irritable Bowel Syndrome, Functional Gastrointestinal Disorders, Medically Unexplained Symptoms, NICE Guideline, Primary Care, General Practitioners

## Abstract

**Background:**

The estimated prevalence of irritable bowel syndrome (IBS) is 10%. Up to one third of patients develop chronic symptoms, which impact on everyday functioning and psychological wellbeing. Guidelines suggest an increased role for primary care in the management of patients with IBS, and referral for psychological interventions. Literature reports dissatisfaction and frustration experienced by both patients with IBS and healthcare professionals. The aim of this study was to explore the perspectives of general practitioners (GPs) in relation to the diagnosis and management of IBS and their views on the potential use of a risk assessment tool to aid management decisions for patients with IBS in primary care.

**Methods:**

This was a qualitative study using face-to-face semi-structured interviews with GPs in North West England. Interviews were fully transcribed and data analyzed using constant comparison across interviews. Tensions between GP accounts and the NICE guideline for the management of IBS were highlighted.

**Results:**

GPs described IBS as a diagnosis of exclusion and the process as tentative and iterative, with delay in adding a Read code to the patient record until they were confident of the diagnosis. Whilst GPs accepted there was a link between IBS and psychological symptoms they suggested that the majority of patients could be managed within primary care without referral for psychological interventions, in conflict with the NICE guideline. They did not feel that a risk assessment tool for patients with IBS would be helpful.

**Conclusions:**

This study highlights the tensions between evidence recognizing the need to identify patients whose symptoms may become chronic and offer pro-active care, including referral for psychological therapies, and the perspectives of GPs managing patients in every-day clinical practice. The reluctance of GPs to refer patients for evidence-based psychological treatments may have implications for commissioning services and patient care.

## Background

Irritable bowel syndrome (IBS) is the commonest of the functional gastrointestinal disorders [1], with an estimated prevalence of 10% [2]. It is a chronic, relapsing and often life-long disorder, characterized by the presence of abdominal pain/discomfort associated with defaecation, a change in bowel habit, the sensation of abdominal distension, and may include associated non-colonic symptoms. As with other ‘medically unexplained symptoms’ (MUS) people with persistent symptoms of IBS have poor physical function, impaired quality of life and high healthcare costs [3]. There is evidence of impairment of health-related quality of life (QOL) [4,5] and qualitative studies have highlighted how symptoms impact on daily functioning, personal and social relationships, self-image and psychological well-being [6,7]. The main predictors for chronicity are psychological distress, symptom duration and disruption of social activities [8]. Factors associated with persistent symptoms are number and severity of symptoms at baseline [9,10].

The majority of patients with IBS are managed within primary care [11] and of those who consult their GP, it has been estimated that up to a third will go on to develop chronic symptoms [8]. The NICE (National Institute for Health and Care Excellence) guideline (CG61 2008) [12] emphasizes establishing a positive diagnosis, identifying symptoms that require prompt referral, but avoiding unnecessary investigations and referrals and working in partnership with the person with IBS. The guideline outlines the evidence for the effectiveness of psychotherapy, cognitive behavioural therapy (CBT) and hypnotherapy in those patients whose symptoms do not respond to pharmacological treatments after 12 months; and emphasizes that the partnership between patient and GP is key, using shared decision-making to aid symptom control [12].

Patients with IBS disproportionately use primary and secondary care services, with sufferers consulting GPs more frequently than matched controls [4,13]. There is evidence of dissatisfaction and frustration voiced by patients and doctors alike. This arises from uncertainties in aetiology and the contested nature of the diagnosis [14], ineffective treatments and a mismatch between GP and patient explanatory models [7,15]. In turn, this can lead to negative stereotyping of patients with IBS by doctors [7,14] and for patients, it may lead to a breakdown in trust and disengagement from services [6,16].

Bijkerk et al. (2003) report that patients and GPs share similar views on aetiology and symptomology, but differ in treatment approaches found acceptable [17]. Dixon-Woods and Critchley (2000) report that GPs can hold hostile views about patients with IBS who are frequent attenders and do not improve [14]. There is little previous work on how a diagnosis of IBS is made in primary care and how this label is applied. Yale et al. (2008) reported that only a small proportion of IBS cases, as recorded in medical records, met case definition criteria, suggesting that diagnosis in primary care may be problematic [18].

This qualitative study was nested in an NIHR (National Institute for Health Research) programme of work (RP-PG-0407-10136) in which one aim was to validate prospectively a risk assessment tool which was developed following an investigation of the predictors of persistent gastrointestinal symptoms amongst new presenters to primary care [8]. The aim of the tool is to identify which patients presenting with a new episode of IBS will go on to develop persistent symptoms, and may therefore potentially benefit from referral for more intensive management, including psychological therapies. The participating practices were not expected to use the tool, but recruit patients to the study.

The aim of the study presented here was to explore qualitatively how GPs currently diagnose and manage IBS, whether management described is consistent with the NICE guideline [12], and GP attitudes towards a predictive, risk assessment tool to aid management and referral decisions in primary care.

## Methods

### Design

Face-to-face, in-depth, semi-structured interviews were conducted with GPs in Greater Manchester, England who were participating in a larger programme of work which aimed to validate prospectively a risk assessment tool for predicting chronicity in patients with IBS. Initially we approached all practices that had a Service Level Agreement with the Greater Manchester Comprehensive Local Research Network (CLRN). We then took a staged approach to roll the study out to general practices within Primary Care Organisations in Greater Manchester. The total number of practices recruited to the study was 58. At the time of recruitment practices were informed about both the Risk Assessment Study and the qualitative interview study. GPs were invited to contact us if they were interested in taking part in the qualitative study. The role of the practices was to recruit patients to the study, rather than use the predictive tool. Data were collected between March and December 2011.

### Sampling

Purposive sampling was used to maximise the variation of the sample of GPs. GPs who were invited to participate in the main study were also invited to participate in this nested qualitative study and two GPs agreed. The remaining 17 GPs were recruited from the 58 practices who had agreed to recruit patients for the main study. We sampled practices according to location, and asked if one of the GPs would participate in the interview study. As recruitment continued, we tried to ensure a spread of participants across gender, ethnicities, age ranges and years of experience in practice. Recruitment was then continued until theme saturation was achieved.

### Data collection

Interviews were conducted, with written consent, at GPs’ premises, by two of the authors (EFH and VH). Interviews lasted between 20 and 70 minutes (mean 42 minutes). The topic guide was developed from review of the existing literature and discussion within the research team. The topic guide, designed to be used flexibly, was modified in the light of emerging themes. The main areas explored were GPs’ views on the aetiology of IBS, how they make the diagnosis of IBS and explain this to patients, treatments offered to patients, and the potential usefulness of a tool to aid their management decisions. The risk assessment tool was described to GPs as a management device to help stratify patients into those who might become high consulters in the long term, and thus may aid future decisions about management of these patients.

Interviews were fully transcribed. Data coding was by constant comparison across interviews [19], by individual authors (CCG, EFH, VH and SH) and emerging themes were agreed through discussion amongst all authors, from different professional backgrounds (academic primary care, health services research, epidemiology). An iterative approach to data collection and analysis was taken; coding and conceptual categories were constantly reviewed and refined in the light of new interview data and ongoing discussion in the research team. Tensions between GP descriptions of their management and the NICE guideline were particularly investigated in the analysis. The topic guide was modified to allow for further exploration of emerging themes. N Vivo 9 (QSR International Pty Ltd, Victoria, Australia) was used to store and manage the data.

## Results

Nineteen interviews with GPs were conducted (see Table 1); 17 GPs had agreed that their practice would recruit patients for the larger programme, two GPs had agreed to be interviewed but their practices declined to recruit patients for the main study. The following themes are presented: an accepted illness, categorizing the patient, diagnosing and labeling, approaches to management, and the utility of a predictive tool in decision-making. Data are presented verbatim from transcripts and identified by a code attributed to the respondent.

**Table 1:** Details of GPs interviewed

		**N**	
Gender	Male	11	
	Female	8	
Age group	30-39	7	
	40-49	5	
	50+	7	
Ethnic origin	White	13	
	Asian/ Asian British	5	
	Black/ Black British	1	
Status	Principal	17	
	Salaried	2	
Mean (range) years in practice		15.4	(4 to 28)
Type of practice	Inner City	2	
	Urban	9	
	Suburban	8	

### An accepted illness

All GPs recognized the existence of IBS and suggested that the diagnosis is not contentious:

*‘I do think it exists, I really do. I think people's lives can be made - it's a label isn't? People who have got constipation predominant IBS might - you could just call them constipated. It's a real condition, people get a lot of pain from it. There are people that seem to get more anxious about it. But we all get a dodgy tummy if you've got an exam or something like that. So I definitely think it exists.’* GP11

All GPs described IBS, however, as a complex condition with a biopsychosocial aetiology, with patients presenting symptoms related to ‘stress’ and lifestyle which could be difficult to untangle:

*‘I think there's a lot of psychology with irritable bowel, not that necessarily the psychology causes the irritable bowel but I think for a fairly benign condition it can cause a lot more upset than you'd expect.’* GP9

GPs suggested that most patients are ready to accept the link between psychosocial issues and bowel symptoms, and may even have considered this before consulting:

‘*Whereas if I involve them throughout the process right from the beginning, er, they adhere to it. I haven't had a single problem with bringing up psychological issues with any of my patients, yeah.*’ GP13

Some GPs alluded to their own experiences, which made symptoms in patients more real, understandable and perhaps a more acceptable diagnosis to make:

*‘I think I’ve dealt with it so much, I’ve experienced it as well and so I think when you’ve experienced it you know how severe some of the symptoms can be.’* GP11

### Categorizing the patient

Most GPs recognised the chronicity of IBS and some suggested that there were a minority of patients who did not improve, attended frequently and were difficult to manage:

‘*I suspect to patients it's a complete nightmare cos it's the daily thing of living with it, but in general, I have to say my experience is that we can help patients but, you know, to improve their quality of life enormously is not necessarily that easy with irritable bowel.*’ GP9

Only a minority of GPs felt there was little they could offer to patients with IBS:

‘*It’s only really if it’s…if there’s no obvious trigger and if it’s causing significant impact on their life. That’s when it becomes very challenging because I think it’s relatively limited what you can do.*’ GP6

GPs recognized that other patients do not return and attributed this to patients either being satisfied with the management offered, or that they simply ‘put up with’ their symptoms:

‘*But sometimes they just never come back and then you just assume they've got better* [laughter].’ GP14

‘*Whether it’s that they just manage it or whether it’s just that we don’t ask them about it any more and they just put up with it I don’t know.*’ GP8

It was thought that only a minority of patients would become frequent attenders:

‘*I mean there are, I guess a sub group of patients with IBS who are the real sort of somatisers I guess, the real patients that, erm, could be termed doctors heart sink patients, erm, so patients that, that are generally very anxious but are anxious about their health, they’re anxious about every little symptom. There is a small group of patients with IBS who fit into that group and so that group of patients have certain patterns, but I wouldn’t say that all patients with IBS are like that.*’ GP18

### Diagnosing and labeling

GPs suggested that the first step in making the diagnosis and managing people with IBS is ensuring that the patient’s concerns are listened to:

‘…*it helps just to reassure the patient that you’re taking the symptoms seriously. I think that’s probably the biggest worry they have, because when you have some functional symptom and you’ve nothing to show the doctor, it’s almost inevitable that you’re going to wonder whether he’ll take it seriously…*’ GP3

Most GPs were aware of the NICE guideline [12] for IBS but only a minority of GPs suggested using the guideline [12] to assist them in managing a patient:

*‘I am aware of them, yeah I am aware of them, the problem with the NICE guidelines is there's millions of them and it's absolutely impossible in normal general day to day practice to be au fait with them all. I would look at them if I really was struggling with managing a patient’.* GP9

The NICE guideline suggests that the diagnosis of IBS should be made in a positive manner considering the symptoms which pointed towards the diagnosis: many GPs felt uncomfortable with this approach, and although few described referring to secondary care to obtain a diagnosis unless there were clear ‘red flag’ symptoms, they described IBS as a diagnosis of exclusion, and the diagnostic process as tentative and iterative, by exclusion of sinister symptoms, the approach taught at medical school:

*‘So to a certain extent there’s always a degree of diagnostic uncertainty because… although I know the term isn’t really favoured any more, but pretty much it’s a diagnosis of exclusion.’* GP18

‘*So now it’s very difficult, yeah, I read the NICE guidelines and it is about positive, it’s not a diagnosis of exclusion any more, it’s about positive symptoms. I’ve read all that but it’s still very hard to go away from something that was drummed in.*’ GP11

Making a diagnosis based on the patient response to medication was also described:

‘…*and, you know, their full blood count would be alright, their coeliac screen would be negative, erm…and then we'd…you know, I'd often give them a trial of Mebeverine or something and that quite often does help, and then they just stay on that.’* GP14

GPs reported that they did not initially add a Read code for IBS to the patient record, but delayed until they were more confident in the diagnosis. Thus, codes such as ‘abdominal pain’, ‘diarrhoea’ or ‘constipation’ would remain on the patient record, rather than IBS, and patients who do not return with their gastrointestinal symptoms would not be coded as having IBS:

‘*I’d probably put a symptom as the coding on the computer at that stage; the most predominant symptom, but if the patient has come in with the same sorts of things over and over again and it’s looking very much like IBS, and it responds to IBS type treatment, then I’d code it.*’ GP1

### Approaches to management: NICE-compliant?

All GPs suggested that giving lifestyle advice, predominantly about diet, to help the patient self-manage their condition, was the first step in management, although most conceded that patients had already tried to modify their diet before consulting:

*‘Some patients have already identified it – ‘My problems seem far worse when I eat such and such…’ so then we say hang on, why don’t you just not eat such and such and see how you do and if they feel that their symptoms have been looked into adequately enough, and they’re reassured that it’s IBS then they’re happy to do that.*’ GP18

Some GPs suggested that a focus on the related psychological symptoms within early consultations might lead to improvement in gastrointestinal symptoms:

‘*Then I tend to sort of not label them as IBS straightaway, yeah. I will manage the stress, I will manage their anxiety, I'll manage the depression and see what happens with the symptoms.*’ GP13

Despite acknowledging the link between psychological symptoms and IBS, all GPs were reluctant to refer patients with IBS for psychological therapies, which is against the recommendation in the NICE guideline [12]. GPs described such primary care mental health services as scarce resources with long waiting times, so they felt the need to reserve these interventions for those patients who they felt had more overt mental health symptoms, rather than a condition such as IBS:

‘*We can refer to the Mental Health team for the depression, with the hope that it might help the IBS symptoms at the same time. We couldn't refer primarily for the IBS.’* GP12

Although psychological therapies such as CBT and hypnotherapy are recommended interventions for IBS in the NICE guideline [12] some GPs expressed doubt about the evidence-base for these interventions:

‘…*the NHS does need to be careful about where it puts its money because…, perhaps we ought to be putting the money into more evidenced based things*.’ GP12

‘*I need to be sort of…I’m not quite sure of the link between why CBT might work, the connection between the psychological component and the patient’s IBS, and CBT. I’m assuming there are studies to show that it does work?’* GP2

GPs recognized that many patients sought help from complementary therapists and either encouraged this or at least did not discourage this approach, which is not advocated by the NICE guideline:

*‘…what I would call a functional bowel disorder and helping manage triggers, a bit, in terms of diet and lifestyle and looking at stress and if they…you know, if they’re doing that and they want to do a bit of homeopathy, as well, okay.’* GP19

One GP described delivering acupuncture to patients with IBS:

*‘Again it’s a balance between getting… being swamped with IBS patients…I told you about a patient who I knew as a friend who, erm, had IBS and he had leakage and when he used to go running, he was very fit, you know, and he used to soil himself I suppose and he tried all medication and I acupunctured him and it stopped him overnight.’* GP16

### Utility of a predictive tool in management and referral

Despite some recognition that there might be a group of patients who may become frequent attenders, GPs did not feel that a risk assessment tool to predict which patients might become high users of care, and thus might benefit from early intervention, would have any utility particularly given the perceived lack of availability of any treatments or referral options:

‘*But don’t we intervene anyway? It’s not as if we sit there doing nothing unless we think someone’s going to be a high consulter, so what actual outcome is that going to have to me and my practice?*’ GP6

Other GPs expressed concern about the practicalities of administering such a tool in a time-limited primary care consultation:

‘*…I can't be doing with having piles of bits of paper, every speciality has got dozens of bits of paper like this and we work across every single speciality, so having bits of paper in the room is a complete loss.*’ GP4

‘…*they’re a pain though, using questionnaires in consultations because you have to find it, you have to print it off if you’ve got it on your computer, if you give it to your patient they then…you can send them away to fill it in but if they fill it in there and that’s in a ten minute consultation.*’ GP11

So the value of a tool to predict which patients might benefit from early intervention for their abdominal symptoms, and direct management decisions, was perceived to be limited to GPs.

## Discussion

### Summary of main findings

This study illustrates how the perspectives of GPs about the diagnosis and management of IBS influence their views on the value of psychological interventions and a risk assessment tool to predict chronicity. GPs accepted that IBS was a legitimate diagnosis to make, although all described reverting to past learning and making the diagnosis by exclusion of sinister symptoms, rather than working with the patient’s positive symptoms suggestive of IBS. In addition, they described reluctance to add the Read code for IBS to a patient’s record, and reflected that many patients with symptoms suggestive of IBS would never receive this label. GPs suggested that most patients with IBS were not difficult to manage; they acknowledged the link between IBS and psychological distress but were reluctant to refer for therapies such as CBT or hypnotherapy, which are advocated by the NICE guideline [12], and did not see the value of a risk assessment tool to predict chronicity and to define who may benefit from such psychological interventions. Most GPs suggested that patients did not return because their symptoms had settled, rather than because they may be dissatisfied with care.

### Strength and limitations of the study

Data are presented from interviews with GPs over a wide geographical area; this purposive sampling enabled us to access a range of views. However, because we employed a theoretical sampling approach, the proportion of GPs holding different views cannot be inferred. Seventeen out of the 19 GPs interviewed were recruiting patients to a study attempting to evaluate the usefulness of a risk assessment tool for IBS in primary care, and were possibly more likely to feel comfortable managing IBS in primary care. These GPs were not using the predictive tool themselves. Furthermore, the proportion of GPs with patients who suffered prolonged or severe forms of IBS is likely to be limited, and in these instances GPs may be more likely to seek external help to support management.

The two GPs who agreed to be interviewed but not to participate in the main study might have been expected to have more negative views about IBS, however this was not apparent in the data.

GPs understanding of the Risk Assessment Tool may have been limited, this was often introduced towards the end of the interview and the way it was presented to respondents could have been different, for example the use of vignettes may have led to a better understanding and discussion of the aims of the risk assessment tool.

Data were collected and analyzed by researchers from different professional backgrounds, thus increasing trustworthiness of the analysis [20].

### Comparison with existing literature

The diagnosis of MUS is often contentious [21], however GPs in this study accepted the existence of IBS, and reported that they had no difficulty in making the diagnosis, or in the management of patients with IBS. These findings are similar to those of Thompson et al. (1997) who reported that compared with pelvic and back pain, IBS was not considered difficult in terms of distinguishing functional from organic disease [22]. When GPs experienced IBS themselves, or knew someone with IBS, it helped them to understand the condition, resonating with Chew-Graham et al. (2008) who described a similar finding with GPs who had first-hand experience of Chronic Fatigue Syndrome [23].

Respondents recognized the link between IBS and psychological distress [11,17,22,24], though they were uncertain of the benefit of psychological therapies in patients with IBS, despite recent evidence of their effectiveness [25,26]. It has previously been reported that GPs in the UK do not refer patients with IBS for psychological treatment [15,22], whereas doctors in the Netherlands are more likely to do so [15]. This is in direct contradiction to the NICE guideline [12] which recommends referral for patients for psychological treatments if symptoms have been present for 12 months or are refractory to treatment. The NICE guideline [12] recommends annual review of patients with IBS, but no GPs reported offering this. In addition, the guideline suggests that patients should be discouraged from seeking complementary therapies such as acupuncture and reflexology. Some GPs in this study reported giving the message that such therapies could be helpful, with one GP delivering acupuncture to selected patients with IBS, in contradiction to the NICE guideline [12].

Previous literature suggests that the diagnosis of IBS is made by excluding red flag indictors, and our study supports this. Hungin et al. (2003) reported that 19% of patients formally diagnosed with IBS had been given the diagnosis on their first visit, and 56% after a further 1–5 visits [27]. This contrasts with a study in which 72% of GPs considered that they were usually or often able to diagnose IBS at the initial visit [22]. These studies were conducted prior to the introduction of the NICE guideline for the diagnosis and management of IBS [12]. However, although most GPs in our study were aware of the guideline, which recommends making a positive diagnosis considering the presence of symptoms, most described an iterative approach to diagnosis with emphasis on exclusion of ‘red flag’ symptoms and described concern about missing something more serious. Thus GPs were reluctant to apply a label of IBS to patients and Read codes were reported as only being applied if the patient re-consults (which they often do not, [6,16]) perhaps explaining differences in prevalence rates of IBS in the literature [27,28], and probably reflecting the fluctuating nature of the condition [29]. This approach has implications for the management of other MUS, particularly those which are less acceptable, for example Chronic Pelvic Pain (CPP) [21], and for GP training about these conditions.

GPs emphasized the importance of self-management in patients with IBS, and felt that offering advice and reassurance was important in the first instance. Dietary advice was emphasized by our respondents, but Bijkerk et al. (2003) reported that dietary advice was not appreciated by patients, who were likely to have already tried such measures before consulting [17].

A strong physician-patient relationship and empowering explanations given by GPs are reported to be important to the successful management of IBS [1,30]. However, Casiday et al. (2008) found that GPs often gave the problem back to the patient [15]. Dixon-Woods et al. (2000) reported that healthcare professionals tended to distinguish between ‘good’ and ‘bad’ patients; with so-called ‘bad’ patients being unaccepting of the diagnosis of IBS, recurrent attenders, demanding of further investigations, failing to cope or respond to treatment, and resentful of the IBS label and psychological explanations given [14]. ‘Good patients’, on the other hand, were those who GPs suggested had a sense of relief with the label given and were accepting of the diagnosis [14]. In the current study, GPs suggested that most patients did not return, attributing this to patients either getting better or learning to live with their symptoms. However, there is evidence to suggest that patients with IBS disengage from services because of dissatisfaction with GP interactions [16], a belief that there is little the NHS can offer, or attributing the onset or worsening of symptoms to previous poor medical care [6].

Of those who consult their GP with a functional gastrointestinal disorder approximately 30% go on to develop chronic symptoms [8]. Robinson et al. (2006) demonstrated that provision of a self-help guidebook, designed with the aid of patients, reduced primary care consultations by 60% [31]. In addition, for patients with intractable symptoms resistant to conventional medical therapy, there may be therapeutic benefit from CBT or hypnotherapy [25,26]. Thus, a risk assessment tool may aid GP decisions about the management of patients at risk of poorer outcomes and fast-track them to therapies that are known to be effective, such as CBT or hypnotherapy, leading to lower levels of distress and healthcare utilization in the long term. Despite most GPs agreeing to recruit patients to the validation study of the risk assessment tool, the majority of GPs interviewed did not feel that a risk assessment tool would be useful to them, whilst others felt that the administration of such a tool would be impractical in a ten-minute consultation.

## Conclusions

Parallels can be drawn with the use of the PhQ-9 where GPs are less receptive of a tool than patients [32], and the value of a tool based on prognostic information to determine the appropriate intervention for patients with back pain that has been shown to be effective [33,34]. Despite the lack of enthusiasm for a risk assessment tool for IBS by GPs, patients might be appreciative of such a tool within the consultation; it may show that the doctor is taking their symptoms seriously and demonstrate patient involvement in decision-making about treatment. This would need to be explored further.

Although the NICE guideline [12] clearly recommends the use of psychological interventions such as CBT and hypnotherapy, for people with chronic symptoms of IBS, the reluctance of GPs to refer patients for such therapies may mean that clinical commissioning groups, led by GPs, are unlikely to commission psychological services for people with IBS, and patients do not receive effective care.

### Ethics approval

The study was approved by NRes Committee North West 5 – Haydock Park.REC Ref No: 09/H1010/38

## Competing interests

This study is part of a wider study to test the validity of a risk assessment questionnaire in patients with IBS.

## Authors’ contributions

EFH participated in the study design, data collection and analysis, and contributed to writing the paper. VH participated in data collection and analysis, and contributed to writing the paper. SH contributed to data analysis and writing the paper. SJO’B contributed to data analysis and writing the paper. DGT contributed to data analysis and writing the paper. PB contributed to study design, participant recruitment and writing the paper. CCG designed the study, supervised data collection and analysis, and led writing the paper. All authors read and approved the final manuscript.

## Authors’ information

CCG, DGT, EFH, SH and VH were supported by the University of Manchester, Faculty of Medical & Health Sciences and the Manchester Academic Health Science Centre (MAHSC) at the time of the study.

## Pre-publication history

The pre-publication history for this paper can be accessed here:

http://www.biomedcentral.com/1471-2296/14/92/prepub

## References

[B1] Chang L Review article: epidemiology and quality of life in functional gastrointestinal disorders Aliment Pharmacol Ther 2004 20 Suppl 7 31 39 15521853 10.1111/j.1365-2036.2004.02183.x

[B2] Wilson S Roberts L Roalfe A Bridge P Singh S Prevalence of irritable bowel syndrome: a community survey Br J Gen Pract 2004 54 495 502 15239910 PMC1324800

[B3] Katon W Lin E Von KM Russo J Lipscomb P Bush T Somatization: a spectrum of severity Am J Psychiatry 1991 148 34 40 1984704 10.1176/ajp.148.7.A34

[B4] Akehurst RL Brazier JE Mathers N O'Keefe C Kaltenthaler E Morgan A Platts M Walters SJ Health-related quality of life and cost impact of irritable bowel syndrome in a UK primary care setting Pharmacoeconomics 2002 20 455 462 10.2165/00019053-200220070-00003 12093301

[B5] Halder SL Locke GR III Talley NJ Fett SL Zinsmeister AR Melton LJ III Impact of functional gastrointestinal disorders on health-related quality of life: a population-based case–control study Aliment Pharmacol Ther 2004 19 233 242 10.1111/j.0269-2813.2004.01807.x 14723614

[B6] Farndale R Roberts L Long-term impact of irritable bowel syndrome: a qualitative study Prim Health Care Res Dev 2011 12 52 67 10.1017/S1463423610000095 21426615

[B7] Casiday RE Hungin AP Cornford CS de Wit NJ Blell MT Patients' explanatory models for irritable bowel syndrome: symptoms and treatment more important than explaining aetiology Fam Pract 2009 26 40 47 19011174 10.1093/fampra/cmn087

[B8] Halder S Macfarlane GJ Thompson D O'Brien SJ Musleh M McBeth J Predictors of persistent gastrointestinal symptoms among new presenters to primary care Eur J Gastroenterol Hepatol 2010 22 296 305 10.1097/MEG.0b013e32832bab61 20169655

[B9] Rief W Rojas G Stability of somatoform symptoms–implications for classification Psychosom Med 2007 69 864 869 10.1097/PSY.0b013e31815b006e 18040096

[B10] Olde Hartman TC Borghuis MS Lucassen PL van de Laar FA Speckens AE van Weel C Medically unexplained symptoms, somatisation disorder and hypochondriasis: course and prognosis. A systematic review J Psychosom Res 2009 66 363 377 10.1016/j.jpsychores.2008.09.018 19379952

[B11] Rubin G de Wit N Meineche-Schmidt V Seifert B Hall N Hungin P The diagnosis of IBS in primary care: consensus development using nominal group technique Fam Pract 2006 23 687 692 10.1093/fampra/cml050 17062586

[B12] National Institute for Health and Clinical Excellence NICE clinical guideline 61: Irritable bowel syndrome in adults: diagnosis and management of irritable bowel syndrome in primary care 2008 London: NICE

[B13] Donker GA Foets M Spreeuwenberg P Patients with irritable bowel syndrome: health status and use of health care services Br J Gen Pract 1999 49 787 792 10885081 PMC1313528

[B14] Dixon-Woods M Critchley S Medical and lay views of irritable bowel syndrome Fam Pract 2000 17 108 113 10.1093/fampra/17.2.108 10758070

[B15] Casiday RE Hungin AP Cornford CS de Wit NJ Blell MT GPs' explanatory models for irritable bowel syndrome: a mismatch with patient models? Fam Pract 2009 26 34 39 19011171 10.1093/fampra/cmn088

[B16] Dhaliwal SK Hunt RH Doctor-patient interaction for irritable bowel syndrome in primary care: a systematic perspective Eur J Gastroenterol Hepatol 2004 16 1161 1166 10.1097/00042737-200411000-00013 15489576

[B17] Bijkerk CJ de Wit NJ Stalman WA Knottnerus JA Hoes AW Muris JW Irritable bowel syndrome in primary care: the patients' and doctors' views on symptoms, etiology and management Can J Gastroenterol 2003 17 363 368 12813601 10.1155/2003/532138

[B18] Yale SH Musana AK Kieke A Hayes J Glurich I Chyou PH Applying case definition criteria to irritable bowel syndrome Clin Med Res 2008 6 9 16 10.3121/cmr.2008.788 18591372 PMC2442028

[B19] Miles M Huberman A Qualitative data analysis: an expanded sourcebook 1994 London, UK: Sage

[B20] Henwood KL Pidgeon NF Qualitative research and psychological theorizing Br J Psychol 1992 83 Pt 1 97 111 1559146 10.1111/j.2044-8295.1992.tb02426.x

[B21] McGowan L Escott D Luker K Creed F Chew-Graham C Is chronic pelvic pain a comfortable diagnosis for primary care practitioners: a qualitative study BMC Fam Pract 2010 11 7 10.1186/1471-2296-11-7 20105323 PMC2835666

[B22] Thompson WG Heaton KW Smyth GT Smyth C Irritable bowel syndrome: the view from general practice Eur J Gastroenterol Hepatol 1997 9 689 692 10.1097/00042737-199707000-00008 9262978

[B23] Chew-Graham CA Cahill G Dowrick C Wearden A Peters S Using multiple sources of knowledge to reach clinical understanding of chronic fatigue syndrome Ann Fam Med 2008 6 340 348 10.1370/afm.867 18626034 PMC2478494

[B24] Johansson PA Farup PG Bracco A Vandvik PO How does comorbidity affect cost of health care in patients with irritable bowel syndrome? A cohort study in general practice BMC Gastroenterol 2010 10 31 10.1186/1471-230X-10-31 20233451 PMC2847968

[B25] Webb AN Kukuruzovic RH Catto-Smith AG Sawyer SM **Hypnotherapy for treatment of irritable bowel syndrome** Cochrane Database Systematic Reviews 2007 4 Art No,CD005110 10.1002/14651858.CD005110.pub2 PMC13178728 17943840

[B26] Zijdenbos IL de Wit NJ van der Heijden GJ Rubin G Quartero AO Psychological treatments for the management of irritable bowel syndrome Cochrane Database Syst Rev 2009 1 CD006442 10.1002/14651858.CD006442.pub2 19160286

[B27] Hungin AP Whorwell PJ Tack J Mearin F The prevalence, patterns and impact of irritable bowel syndrome: an international survey of 40,000 subjects Aliment Pharmacol Ther 2003 17 643 650 10.1046/j.1365-2036.2003.01456.x 12641512

[B28] Talley NJ Irritable bowel syndrome: definition, diagnosis and epidemiology Baillieres Best Pract Res Clin Gastroenterol 1999 13 371 384 10.1053/bega.1999.0033 10580915

[B29] Williams RE Black CL Kim HY Andrews EB Mangel AW Buda JJ Cook SF Stability of irritable bowel syndrome using a Rome II-based classification Aliment Pharmacol Ther 2006 23 197 205 10.1111/j.1365-2036.2006.02723.x 16393298

[B30] Guthrie E Medically unexplained symptoms in primary care Advances in psychiatric treatment 2008 14 432 440 10.1192/apt.bp.106.003335

[B31] Robinson A Lee V Kennedy A Middleton L Rogers A Thompson DG Reeves D A randomised controlled trial of self-help interventions in patients with a primary care diagnosis of irritable bowel syndrome Gut 2006 55 643 648 10.1136/gut.2004.062901 16099784 PMC1856107

[B32] Malpass A Shaw A Kessler D Sharp D Concordance between PHQ-9 scores and patients' experiences of depression: a mixed methods study Br J Gen Pract 2010 60 e231 e238 10.3399/bjgp10X502119 20529486 PMC2880764

[B33] Hill JC Whitehurst DG Lewis M Bryan S Dunn KM Foster NE Konstantinou K Main CJ Mason E Somerville S Sowden G Vohora K Hay EM Comparison of stratified primary care management for low back pain with current best practice (STarT Back): a randomised controlled trial Lancet 2011 378 1560 1571 10.1016/S0140-6736(11)60937-9 21963002 PMC3208163

[B34] Sanders T Foster NE Ong BN Perceptions of general practitioners towards the use of a new system for treating back pain: a qualitative interview study BMC Med 2011 9 49 10.1186/1741-7015-9-49 21554696 PMC3098180

